# Intersection of Structural Racism, Social Determinants of Health, and Implicit Bias With Emergency Physician Admission Tendencies

**DOI:** 10.1001/jamanetworkopen.2021.26375

**Published:** 2021-09-01

**Authors:** Leonard E. Egede, Rebekah J. Walker, Joni S. Williams

**Affiliations:** Division of General Internal Medicine, Department of Medicine, Medical College of Wisconsin, Milwaukee; Center for Advancing Population Science, Medical College of Wisconsin, Milwaukee.

The current social and political landscape, shaped by centuries of exclusive and discriminatory practices, decades of compounded experiences with police brutality, residential segregation, and unfair criminal justice proceedings, and recent reactions to large-scale demonstrations and protests, highlights the intersection of structural racism, social determinants of health, and bias experienced by individuals entering the US health care system. The COVID-19 pandemic exposed the magnitude of influence these factors have on the health of racial and ethnic minority communities, uncovering disparities in how individuals receive care and the outcomes of that care.^1^ Khidir and colleagues^2^ investigate implicit bias in emergency department (ED) physician admission tendencies by race, ethnicity, sex, and Medicaid enrollment status of patients. Using a 20% random sample of Medicare claims from 2012 through 2015, the authors assessed whether within-hospital physician-level variation in admission rates differed by sociodemographic characteristics of Medicare enrollees and whether there was implicit bias in admission decisions. Although substantial differences were noted in rates of admission by race, ethnicity, and sex of the enrollees, and variation was seen in tendencies to admit across physicians, the study found consistency in ED physician admission tendencies across sociodemographic groups, and the authors suggest that this finding indicates limited implicit bias in admission tendencies.

The authors are commended on their use of sophisticated methods to investigate a pressing issue; however, there are several areas that suggest the results should be interpreted with caution. First, hospital-level factors were treated as fixed effects, despite earlier research showing varying patterns of care across hospitals.^3^ For example, an investigation of racial differences in ED admission and length of stay found complex differences requiring investigation into both within- and amonghospital components to understand admission disparities.^3^ Aspects of the hospitals, such as location, whether the hospital is academic or community-based, and the demographic make-up of hospital staff and patients, can influence care in a variety of ways. In addition, hospital systems influence physician practice patterns,^4^ and therefore, removing hospitals from the analysis may oversimplify variation and lead to inflated consistency within physicians in the same system. Second, factors possibly affecting physician decision-making were not captured in this study, despite evidence that physician decision-making is influenced by technical skills, training, clinical experience, professional values, and personal attitudes and perceptions.^4^ In addition, the study sample was not representative of the typical ED population, excluding younger-aged individuals and frequent ED users. For example, Khidir and colleagues^2^ used a Medicare sample, was limited to the 37 most frequent medical diagnoses, and excluded ED visits that occurred within 30 days of a previous visit. Therefore, the conclusions may be different with a more typical ED population.

Disparities in health care continue to persist, and factors at the clinician level, such as implicit bias, stereotyping, prejudice, and perceptions based on how patients present, contribute to the observed differences in health outcomes.^5^ Implicit bias occurs when personal attitudes toward patients unconsciously influence understanding, actions, and decisions.^6^ Higher and stronger levels of these implicit biases are associated with worsening patient-clinician interactions and communication, differences in the therapeutic bond, treatment decisions, and recommendations by the clinician or treatment adherence by the patient, and disparities in patient outcomes.^5^ Examples of these patterns in health care delivery include women receiving fewer cardiovascular disease reduction treatments compared with men, individuals of racial and ethnic minorities perceived as having less pain and more medication-seeking behavior than non-Hispanic White individuals, and persons of racial and ethnic minorities with mental health conditions more often diagnosed as having psychosis rather than mood disorders compared with their non-Hispanic White counterparts.^5^ Evidence shows implicit biases exist among ED clinicians and suggests these biases occur as a result of patient load, overcrowding, and cognitive load, resulting from unique time constraints in the ED, mental stressors, lack of care continuity, limited comprehensive clinical data, and variability in patient acuity and concerns.^6^ Furthermore, among large societal factors that contribute to implicit bias, the ED can serve as the source for stereotyping and bias in medical care before hospital admission.^6^ Therefore, further research is warranted to understand the role of implicit bias among clinicians and hospitals on patient- and systems-level outcomes and their contribution to perpetuating disparities.

Decisions to admit patients to the hospital should be based on objective measures, such as comorbidity status, disease severity, and potential for complications; however, evidence from previous research suggests this is not always the case.^5^ There are also lingering and unanswered questions of how bias plays into decision-making regarding who should be admitted and how systemic bias within the health care system plays a role in this process. An important concept often left out of investigations on bias is that of intersectionality, defined by Collins and Bilge as “a way of understanding and explaining complexity in the world, in people, and in human experiences, which are generally shaped by many factors in diverse and mutually influencing ways.”^7,8^ Individuals do not have a single identity, such as race, ethnicity, sex, gender, socioeconomic status, insurance status, or religion, but have multiple social intersections by which they identify.^7^ For example, clinicians may respond differently to an African American woman identifying as transgender compared with a non-Hispanic White woman identifying as heterosexual by asking different questions, which may result in a different decision regarding admission. The individual may not be aware of whether their race, sex, or gender identity influenced the decision to admit, or if an intersection of any of these identities may have biased the questions and thus the decision. Similarly, a man who is Arab American, Muslim, and uninsured, compared with a non-Hispanic White man who is Christian and uninsured, may experience different types of discrimination within the health care system, with few of these types likely a result of only one aspect of their identity. If analyses focus only on the fact both individuals are uninsured and not the multiple forms of social inequality, which converge to influence their health, the result will be a simplified version of reality. Multilevel modeling of intersecting dimensions of an individual may be needed to fully understand the influence of intersectionality on health.^7^

In conclusion, this study should be seen as the beginning of the next phase of research into implicit bias in health care, instead of assuming it answers the question of whether implicit bias exists in the health system.^2^ The authors are applauded for addressing the issue; however, it is premature to assume implicit bias does not exist in physician admission tendencies given the evidence in the literature to the contrary. In addition, to assume differences in rates of admission are the result of social determinants of health ignores the literature on the role of bias in physician decision-making and the contribution of physician- and hospital-level factors on physician decision-making. In light of evidence of the influence of structural racism on health outcomes and emerging evidence on intersectionality, future studies need to capture detailed information on hospital-, physician-, and patient-level factors as well as incorporate intersectionality of patients’ identities to adequately understand factors associated with physician decision-making, including decisions to admit or discharge patients from the ED.
